# Trop2 marks transient gastric fetal epithelium and adult regenerating cells after epithelial damage

**DOI:** 10.1242/dev.131490

**Published:** 2016-05-01

**Authors:** Valeria Fernandez Vallone, Morgane Leprovots, Sandra Strollo, Gabriela Vasile, Anne Lefort, Frederick Libert, Gilbert Vassart, Marie-Isabelle Garcia

**Affiliations:** Institut de Recherche Interdisciplinaire en Biologie Humaine et Moléculaire (IRIBHM), Faculty of Medicine, Université Libre de Bruxelles ULB, Route de Lennik 808, Brussels 1070, Belgium

**Keywords:** Lgr5, Embryonic, Spheroids, Indomethacin, Stomach, Tacstd2

## Abstract

Mouse fetal intestinal progenitors lining the epithelium prior to villogenesis grow as spheroids when cultured *ex vivo* and express the transmembrane glycoprotein Trop2 as a marker. Here, we report the characterization of Trop2-expressing cells from fetal pre-glandular stomach, growing as immortal undifferentiated spheroids, and their relationship with gastric development and regeneration. Trop2^+^ cells generating gastric spheroids differed from adult glandular Lgr5^+^ stem cells, but appeared highly related to fetal intestinal spheroids. Although they shared a common spheroid signature, intestinal and gastric fetal spheroid-generating cells expressed organ-specific transcription factors and were committed to intestinal and glandular gastric differentiation, respectively. Trop2 expression was transient during glandular stomach development, being lost at the onset of gland formation, whereas it persisted in the squamous forestomach. Undetectable under homeostasis, Trop2 was strongly re-expressed in glands after acute Lgr5^+^ stem cell ablation or following indomethacin-induced injury. These highly proliferative reactive adult Trop2^+^ cells exhibited a transcriptome displaying similarity with that of gastric embryonic Trop2^+^ cells, suggesting that epithelium regeneration in adult stomach glands involves the partial re-expression of a fetal genetic program.

## Abstract (precis)

**Summary:** Trop2, a marker of gastric fetal glandular epithelium grown *ex vivo*, is re-expressed upon injury in adult regenerative cells together with a partial fetal-like genetic program.

## INTRODUCTION

Adult epithelia lining the digestive tract rely on a panel of stem cells to self-renew and maintain tissue homeostasis following injury. *In vivo* lineage tracing and the development of methods allowing the stable culture of minigut organoids *ex vivo*, have been instrumental in identification of the various stem cells in the small intestine (Barker et al., 2007; Sato et al., 2009) as well as in the corpus and antrum regions of the glandular stomach (Hoffmann, 2015). Under homeostatic conditions, actively cycling cells behaving as stem/progenitor cells are essentially located in the isthmus of the corpus glands. Their differentiation generates pit and neck cells secreting mucins, parietal cells that produce hydrochloric acid, as well as chief cells and endocrine cells producing zymogens and hormones, respectively (Hoffmann, 2015). Cells expressing *Tff2* transcripts, but not the corresponding protein, have been reported to behave as gland progenitors (Quante et al., 2010). Upon epithelial injury, corpus cells with chief cell characteristics expressing tumor necrosis factor receptor 19 (Tnfrsf19; also known as Troy) can de-differentiate and function as reserve stem cells to repopulate the glands (Nam et al., 2010; Stange et al., 2013). In corpus and antral glands, Sox2 traces progenitors and adult stem cells (Arnold et al., 2011). In the antrum, actively cycling stem cells are present in the bottom of the glands and express leucine-rich repeat G protein-coupled receptor 5 (Lgr5). They give rise mainly to mucus-secreting and endocrine cells (Barker et al., 2010). Moreover, a pool of rare quiescent villin-traced cells has been reported to be reactivated upon interferon gamma treatment, leading to repopulation of entire antral gland units; however, their molecular signature remains unknown (Qiao et al., 2007).

In addition to its use in the identification of adult stem cells from tissues as diverse as intestine, stomach, liver and pancreas (Barker et al., 2010; Huch et al., 2013a,b; Sato et al., 2009), the three-dimensional culture system has recently been used to isolate and characterize epithelial progenitors of the small intestine in the fetus (Fordham et al., 2013; Mustata et al., 2013). In contrast to organoids, with their lineage-specific differentiated cell types mimicking adult tissue, these cells grow *ex vivo* as poorly differentiated immortal hollow spheroids. They retain, however, the potential to convert into adult Lgr5-expressing (Lgr5^+^) intestinal stem cells both *ex vivo* and in grafting experiments after epithelial injury *in vivo* (Fordham et al., 2013; Mustata et al., 2013). These intestinal progenitors are identified by their high expression levels of the cell surface molecule Trop2 [also known as tumor-associated calcium signal transducer 2 (Tacstd2)]. Initially discovered as a marker of invasive trophoblasts, Trop2 expression has also been reported in various organs during development and in adult stem cells during homeostasis, as well as in regenerative conditions and cancer (McDougall et al., 2015; Shvartsur and Bonavida, 2015).

In the mouse stomach, primary specification of the epithelium occurs before embryonic day (E) 11.5, preceding a secondary phase at ∼E15, which leads to the emergence of gastric units in the presumptive glandular region. In the forestomach, a squamous stratified epithelium develops with characteristics similar to that of esophagus. We show here that Trop2 marks fetal glandular epithelial cells of the stomach, growing as spheroids when cultured *ex vivo*. In adults, upon injury, Trop2 expression is reactivated in regenerative cells together with part of a fetal-like genetic program.

## RESULTS

### Gastric Trop2-expressing fetal cells grow as immortal spheroids *ex vivo*

**Fig. 1.**
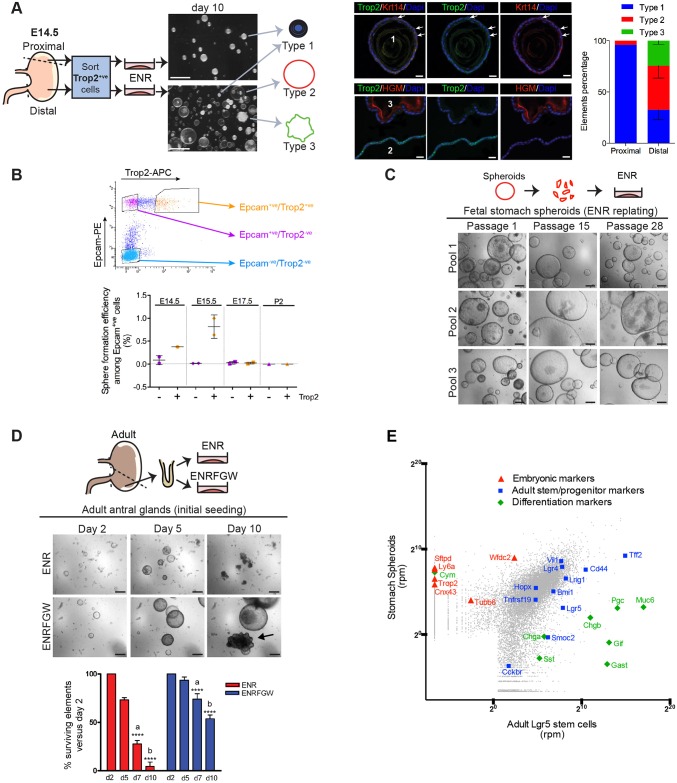
**Gastric Trop2-expressing fetal cells grow as immortal spheroids *ex vivo*.** (A) Spheroid-generating cells originate from pre-glandular stomach. (Left) Sorted Trop2^+^ cells from E14.5 proximal and distal stomach regions were cultured in ENR (EGF, noggin, R-spondin 1) conditions for 10 days. (Right) Immunofluorescence for type 1, 2 and 3 elements showing keratin 14 (Krt14), Muc5ac (HGM antibody) and Trop2 expression. Arrows indicate stratified cells. The proportion of the three types grown from each source is shown far right; mean±s.e.m. (*n*=2 independent sorting experiments). (B) Spheroid formation efficiency among Epcam^+^ cells isolated from distal stomach depends on embryonic stage and Trop2 expression (*n*=2 independent sorting experiments). None of the Epcam^−^ cells generated spheroids. (C) Representative images of fetal stomach spheroids from three different pools during passages. (D) Representative images from initial seeding of adult antral glands under ENR and ENRFGW (ENR plus Fgf10, gastrin, Wnt3a) conditions. The same field was followed over time; arrow shows a differentiating organoid element. Beneath is shown the percentage of surviving elements over time (*n*=4 mice). Two-way ANOVA, ****P<0.0001 for medium/time effect; Bonferroni, a,b *P*<0.0001. (E) Transcriptome analysis of gastric fetal spheroids (three independent pools) and sorted adult Lgr5^+^ stem cells (four mice pooled) presented as a scatter plot of the log_2_ mean. Rpm, reads per million. Genes representing embryonic, adult stem/progenitor or glandular differentiation markers are highlighted. Scale bars: 1 mm in A left; 20 µm in A right; 200 µm in C,D.

Fetal progenitors lining the intestinal epithelium before cytodifferentiation were previously identified as Trop2-expressing (Trop2^+^) cells growing as undifferentiated spheroids when cultured in Matrigel in the presence of EGF, noggin and R-spondin 1 (hereafter referred to as ENR culture conditions) (Mustata et al., 2013). In the present study, we explored whether similar cells could be cultured from the fetal stomach. E14.5 stomachs were divided into two parts: the proximal region, and the rest of the stomach referred to as the distal zone. Trop2^+^ cells were sorted, seeded in Matrigel and cultured in the ENR medium. Trop2^+^ cells from the proximal region generated almost exclusively (98.5%) small round dark elements composed of mono- or multilayered keratin 14-expressing cells characteristic of squamous epithelium (type 1; Fig. 1A). By contrast, the distal samples generated a majority of clear spheroid-like structures, with a morphology reminiscent of fetal intestinal spheroids (49.8%, type 2), together with some organoid-like elements (24.4%, type 3; Fig. 1A) and type 1 squamous contaminants. These spheroids did not express squamous markers, nor the mucous pit cell marker Muc5ac (recognized by HGM antibody). On the other hand, HGM was readily detected in type 3 organoid-like structures, which are likely to correspond to elements engaged in differentiation (Fig. 1A).

Next, the spheroid formation capacity of epithelial (Epcam^+^) cells from the pre-glandular region was studied over time. It was clearly positively correlated with both early developmental stages (E14.5-15.5) and Trop2 expression (Fig. 1B). Of note, the drop in spheroid formation capacity observed between E15.5 and E17.5 implies some degree of heterogeneity among the Trop2^+^ population over time. Altogether, this indicated that spheroids were generated from poorly differentiated transient Trop2^+^ cells coming from the presumptive glandular region. Spheroids could be efficiently replated under ENR conditions for at least 30 passages (5 months), retaining their hollow spheroid morphology (Fig. 1C). Their growth properties differed from those of adult gastric organoids, the culture of which is optimized in a medium further supplemented with Fgf10, gastrin (Gast) and Wnt3a (referred to as ENRFGW) (Barker et al., 2010; Stange et al., 2013). We confirmed that antral glands from adults generate only a limited number of elements able to survive under ENR conditions (Fig. 1D).

The gastric spheroid transcriptome was compared with that of antral Lgr5^+^ stem cells by RNA-Seq analysis (Fig. 1E). Spheroids expressed lower levels of markers reportedly associated with adult gastric stem cells or with facultative stem cells (Lgr5, CD44, Tnfrsf19, Tff2, Cckbr) (Hoffmann, 2015). Since some of these markers are Wnt target genes, this suggests that Wnt signaling activity is lower in fetal spheroids than in adult stem cells. Spheroids also displayed lower expression of glandular differentiation markers for mucous neck, chief, or endocrine cells (Muc6, Pgc, Gif, Chga, Chgb, Sst and Gast). By contrast, they showed higher expression levels of reported intestinal progenitor markers [Trop2, Cnx43 (Gja1), Sftpd, Ly6a, Wfdc2 and Tubb6] (Mustata et al., 2013), together with chymosin (Cym), which is described as a marker of immature stomach (Fig. 1E, Fig. S1A) (Chen et al., 2001). This expression profile was stable over at least 20 passages (Fig. S1B). Altogether, these data identified fetal gastric spheroids as self-renewing elements with a phenotype and growth properties clearly distinct from those of adult Lgr5^+^ stem cells.

### Spheroid-generating cells are committed to a gastric glandular fate

**Fig. 2.**
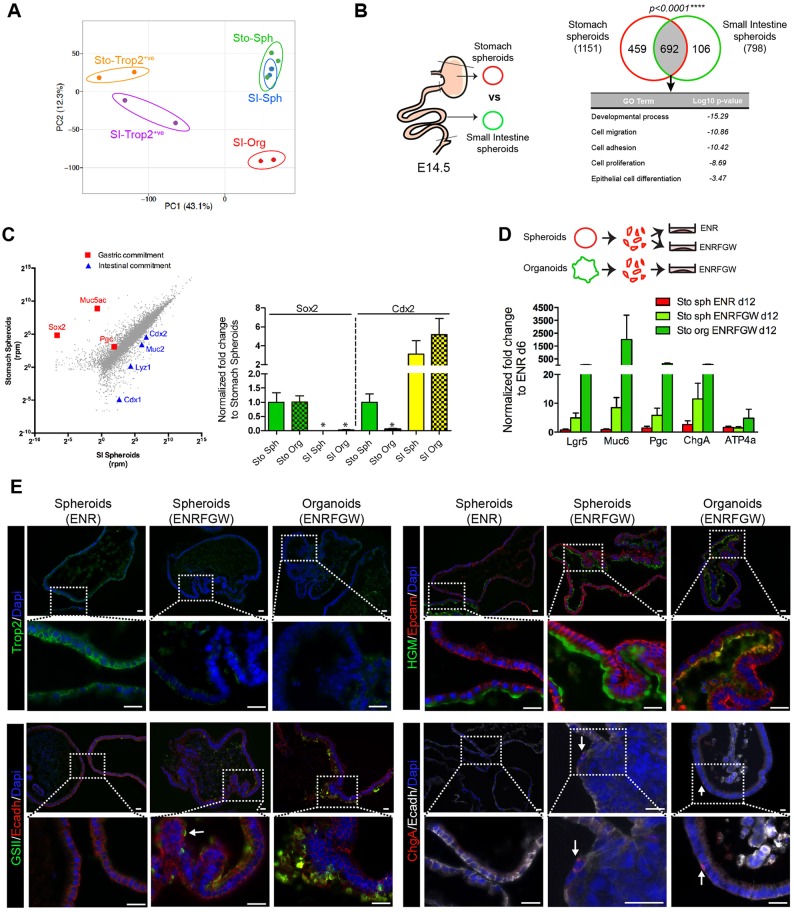
**Spheroid-generating cells are committed to a gastric glandular fate.** (A) PCA plot of transcriptome (19,468 genes) from cultured stomach (Sto-Sph) and intestinal (SI-Sph) spheroids, intestinal organoids (SI-Org) and sorted stomach and intestinal Trop2^+^ cells (Sto-Trop2^+^ and SI-Trop^+^). (B) The Venn diagram shows the overlap between ≥4-fold upregulated genes (number of genes in parentheses) in stomach and intestinal spheroids (both versus intestinal organoids). Chi-square with Yates’ correction. GO term analysis is shown for the common ‘fetal spheroid signature’. (C) (Left) Scatter plot of gastric and intestinal fetal spheroid transcriptomes (three independent pools) presented as the log_2_ mean. Rpm, reads per million. Genes representing gastric and intestinal commitment are highlighted. (Right) *Sox2* and *Cdx2* mRNA expression levels measured by qRT-PCR in stomach spheroids (Sto Sph; *n*=6), stomach organoids (Sto Org; *n*=5), small intestine spheroids (SI Sph; *n*=4) and small intestine organoids (SI Org; *n*=4). Asterisks indicate values below the mean value of 0.05. (D) Stomach spheroid cells can differentiate in ENRFGW medium. qRT-PCR of selected gastric differentiation genes in Sto Sph at day 6 and day 12 (*n*=4), using Sto Org (*n*=3) obtained at day 12 as a positive differentiation control. Mucin 6 (*Muc6*), pepsinogen C (*Pgc*; also known as progastricsin), chromogranin A (*Chga*) and proton pump (*Atp4a*) are markers of mucus neck, chief, endocrine and parietal cells, respectively. Two-way ANOVA, *P*=0.0018 for media effect on Sto Sph. (E) Immunofluorescence showing low and high magnification for Trop2 staining, or cell differentiation markers (HGM, GS-II and ChgA), of spheroids cultured under ENR or ENRFGW conditions, with stomach organoids as positive controls. Membrane Epcam and E-cadherin (Ecadh) stainings help to define epithelial cell limits. Arrows indicate differentiated cells. Scale bars: 20 µm.

As fetal intestinal spheroids were previously found to express several genes belonging to the gastric differentiation program (Mustata et al., 2013), we compared the transcriptome of fetal gastric and intestinal spheroids. Principal component analysis (PCA) plot of all expressed genes showed that intestinal spheroids clustered with gastric spheroids rather than with intestinal organoids (Fig. 2A). By selecting transcripts that were at least 4-fold upregulated in gastric and intestinal spheroids compared with intestinal organoids, we defined a list of 692 commonly upregulated genes as the ‘fetal spheroid signature’ (Fig. 2B). Gene ontology (GO) term analysis of this list revealed a significant correlation with processes related to tissue development, cell migration, adhesion, proliferation and cell differentiation (Fig. 2B, Table S1). Of note, despite the expected divergence between gastric E14.5 Trop2^+^ cells sorted from tissue and cultured spheroid cells (Fig. 2A), both cell types shared expression of embryonic markers, as compared with adult-derived cells (Fig. S2A). This indicated that the global gene expression profile of fetal gastric cells is maintained, at least partly, in cultured spheroids.

Despite the high degree of similarity between gastric and intestinal spheroid transcriptomes, key transcription factors associated with patterning of the foregut (Sox2) and midgut (Cdx1) regions, as well as gastric and intestinal differentiation markers (Muc5ac and Muc2, respectively), were expressed in a tissue of origin-specific manner (Fig. 2C). Unexpectedly, the intestinal Cdx2 factor was also expressed, although at a lower level, in gastric spheroids (Fig. 2C, Fig. S2B).

To investigate the differentiation potential of gastric spheroids *ex vivo*, spheroids serially passaged in ENR conditions were plated in the adult-type ENRFGW culture medium. This led to significant upregulation of the adult stem cell marker *Lgr5* and cell lineage differentiation markers of the stomach glands at the transcriptional level (Fig. 2D). Accordingly, morphologically differentiated mucous neck and pit and endocrine (GS-II^+^, HGM^+^, ChgA^+^) cells were observed, similar to those detected in adult-type organoids (Fig. 2E). Although *Pgc* transcripts were detected, mature chief cells could not be identified morphologically. In addition, shifting spheroids to ENRFGW did not lead to upregulation of the parietal marker *Atp4a* (Fig. 2D). Concomitantly, expression of the embryonic marker Trop2, detected at the membrane level in spheroids, decreased or disappeared in organoid-like structures emerging from spheroid-derived ENRFGW cultures (Fig. 2E). Of note, some morphologically differentiated cells still co-expressed Trop2, suggesting an ongoing differentiation process in these elements (Fig. S2C). Similar differentiation results were obtained in later passaged spheroids (Fig. S2D). No evidence for differentiation towards the intestinal or squamous epithelial types was observed in spheroids cultured in ENR medium (Fig. S2E). Overall, these experiments indicated that, despite their expression of the intestinal Cdx2 transcription factor, Sox2^+^ spheroids derived from the fetal stomach are clearly committed to a gastric glandular fate.

### Transient expression of the Trop2 and Cnx43 markers in pre-glandular epithelial cells

**Fig. 3.**
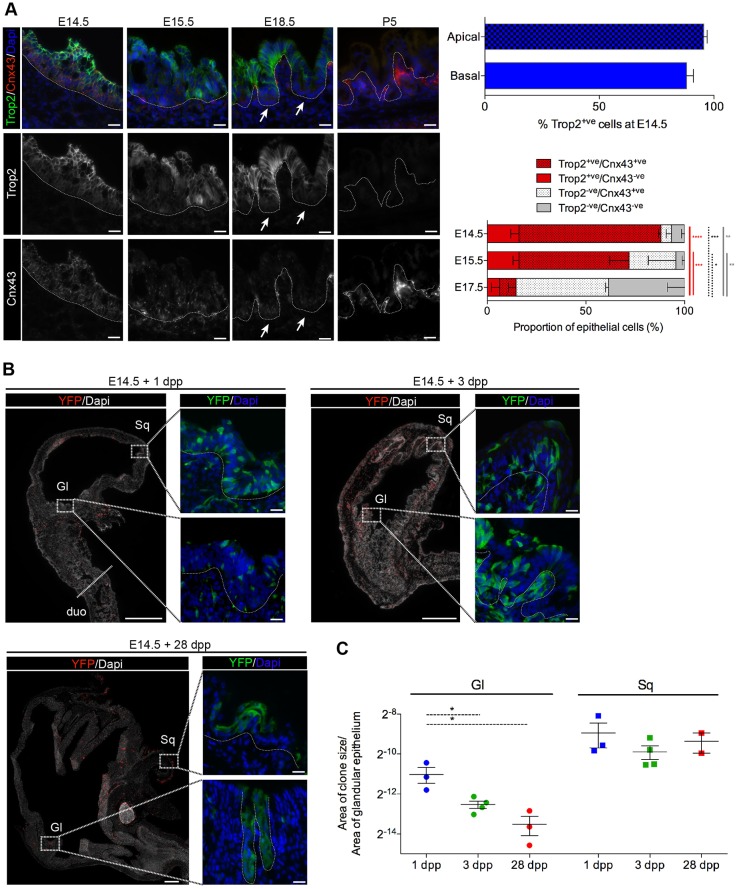
**Transient expression of Trop2 and Cnx43 markers in pre-glandular epithelial cells.** (A) (Left) Immunofluorescence showing expression of Trop2 and Cnx43 during development. Arrows point to absence of Trop2 expression at the bottom of the glands, where positive punctate staining of Cnx43 is still present. (Right) The proportion of apical and basal cells expressing Trop2 (top) and the proportion of cells expressing embryonic markers in the most basal cells (bottom). *n*=3 embryos per time point. Two-way ANOVA, *P<0.05, **P<0.01, ***P<0.001, ****P<0.0001. (B) Representative images for lineage-tracing experiments in Cnx43-CreER/Rosa26R-YFP mice. Low and high magnification of squamous (Sq) and glandular (Gl) stomach; tamoxifen was administrated at E14.5 and analyzed 1, 3 and 28 days post pulse (dpp). duo, duodenum. Dotted lines delineate epithelial/mesenchymal boundaries. (C) Relative proportion of Cnx43-traced clone area per total Gl or Sq epithelial area. Each dot corresponds to a single embryo/mouse. Non-parametric one-way ANOVA, *P<0.05. Scale bars: 20 µm in A; 500 µm (low magnification) and 20 µm (high magnification) in B.

Expression of the spheroid markers Trop2 and Cnx43, identified *ex vivo*, was studied during stomach development in the presumptive glandular region by immunofluorescence. At E14.5, Trop2 expression was detected in most epithelial cells (91.8±2.2%; Fig. 3A). During gland formation (E15.5-18.5), a progressive decrease of Trop2^+^/Cnx43^+^ cells was observed, manifesting mainly in the most basal cells of the developing glandular epithelium (Fig. 3A). This was accompanied, between E14.5 and E17.5, by a concomitant increase in the proportions of proliferating cells displaying Trop2^−^/Cnx43^+^ or Trop2^−^/Cnx43^−^ phenotypes (from 5.4±2.9 to 52.1±1.4% and from 3.4±1.8 to 37.7±7.0%, respectively). In the postnatal period [from postnatal day (P) 5 onward], neither Trop2 nor Cnx43 were detected in the epithelium, with Cnx43 labeling only present in the mesenchyme (Fig. 3A). This transient expression during stomach development was not observed for other epithelial markers, such as the Trop2-related marker Epcam1, which was detected throughout the pre- to postnatal periods (E14.5 to P20) (Fig. S3A). Together, these data indicate the existence of a transient population of Trop2^+^/Cnx43^+^ cells in the fetal glandular epithelium during development. By contrast, in the forestomach, strong expression of Trop2 and Cnx43 was maintained in the different layers of the squamous epithelium throughout development and postnatally (Fig. S3B).

Since the vast majority of epithelial Cnx43^+^ cells (95.1±2.6%) co-expressed Trop2 at E14.5, the fate of Trop2^+^/Cnx43^+^ embryonic cells was followed by lineage tracing using the Cnx43-CreER/Rosa26R-YFP mouse strain (Fig. 3B,C). One day following a tamoxifen pulse (1 dpp) given at E14.5, YFP^+^ cells were detected as sparse small clones in the squamous and presumptive glandular epithelia, as well as in the corresponding mesenchymal compartments. Despite a similar increase in the size of individual clones in both epithelial regions, at 3 dpp and, more so, at 28 dpp, the proportion of labeled cells contributing to the glandular epithelium decreased significantly, whereas it remained stable in the squamous epithelium (Fig. 3B,C, Fig. S3B,C). Together with the observed concomitant increase in proliferating Cnx43^−^ cells, this suggests that E14.5 Trop2^+^/Cnx43^+^ cells contribute mainly to glandular formation during the fetal stages, with a definite but limited contribution to the postnatal epithelium resulting from dilution of the E14.5-traced cells by later progenitors unrelated to the Cnx43-Cre lineage.

### Re-expression of the Trop2 marker in damaged adult stomach

**Fig. 4.**
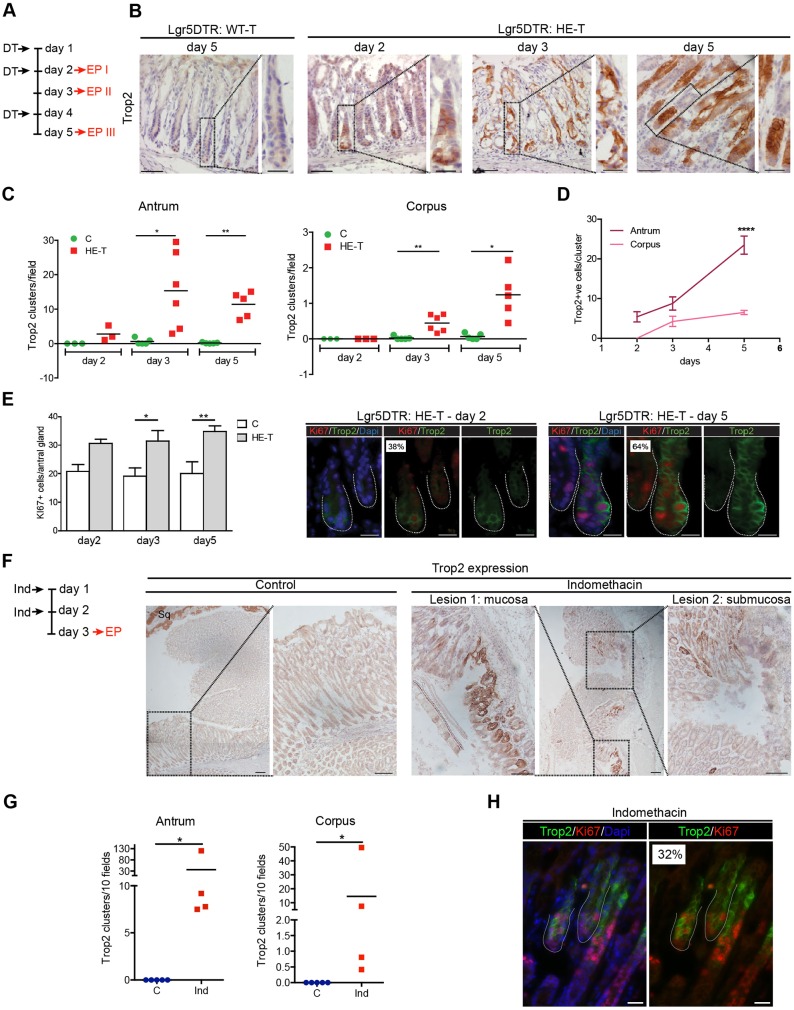
**Re-expression of the Trop2 marker in damaged adult stomach*.*** (A) Design of the experiment for Lgr5^+^ cell ablation in *Lgr5*-DTR mice with diphtheria toxin (DT). EP, endpoint. (B) Immunohistochemistry of antral gland sections showing Trop2 expression after DT treatment of wild-type (WT-T) or heterozygous *Lgr5*-DTR (HE-T) mice. (C) Quantification of Trop2^+^ clusters per field in HE-T and control (label C; non-treated heterozygous *Lgr5*-DTR HE-NT and WT-T) animals. Individual mice are represented by the green circles and red squares. Unpaired *t*-test with Welch's correction; antrum, *P=0.023, **P=0.002; corpus, *P=0.017, **P=0.007. (D) Quantification of the number of Trop2^+^ cells/cluster over time. Mice: *n*=3 at day 2; *n*=4 at day 3 and day 5. Two-way ANOVA, ****P=0.0008. (E) (Left) Quantification of total number of Ki67^+^ cells per antral gland. Mice: *n*=3 at all time points for control; *n*=3, 4 and 7 for HE-T at day 2, day 3 and day 5, respectively. Two-way ANOVA, *P*<0.0001 for treatment effect; Bonferroni, *P=0.03, **P=0.004. (Right) Immunofluorescence showing co-expression of Ki67 in Trop2^+^ cells in HE-T glands. The percentage of Trop2^+^ proliferating cells is indicated (*n*=3 and 4 at day 2 and day 5, respectively). (F) (Left) Experimental scheme for *in vivo* treatment with indomethacin (Ind). (Right) Representative immunohistochemistry showing Trop2 expression in the vicinity of Ind-induced lesions. (G) Quantification of Trop2^+^ clusters/10 fields in Ind-treated and vehicle-treated control animals. Non-parametric Mann–Whitney; antrum and corpus, *P=0.0159. (H) Representative immunofluorescence showing co-expression of Ki67 and Trop2 in glands of Ind-treated mice. The percentage of Trop2^+^ proliferating cells is indicated (*n*=2). Scale bars: 50 µm in B; 20 µm in B insets, E,H; 200 µm (low magnification) and 100 µm (high magnification) in F.

Adult Lgr5^+^ stem cells ensure constant renewal of the stomach epithelium under homeostatic conditions (Barker et al., 2010). To investigate the potential re-expression of embryonic markers in the absence of Lgr5^+^ cells, we induced their specific ablation (Tian et al., 2011) by injecting *Lgr5*-DTR-EGFP mice with diphtheria toxin (DT) (Fig. 4A). As early as 24 h after the first injection (day 2), DT treatment was associated with the appearance of apoptotic cells and concomitant loss of *Lgr5*^+^ cells in the bottom of the glands of *Lgr5*-DTR mice, indicating efficient ablation (Fig. S4A). Significant weight loss was observed in DT-treated heterozygous *Lgr5*-DTR mice (HE-T), but not in control animals [vehicle-treated heterozygous *Lgr5*-DTR (HE-NT) or DT-treated wild-type mice generated in the context of the *Lgr5*-DTR (WT-T)] after the third day of treatment (Fig. S4B). Whereas the spheroid-associated marker Cnx43 was not induced in the epithelium of HE-T mice, strong membrane expression of Trop2 was specifically detected in HE-T glands (Fig. S4C). Trop2^+^ cells were detected as small clusters at day 2 (Fig. 4B). At day 3, a stage associated with an overall disorganization of the HE-T glands as characterized by the presence of many cystic structures, the number of Trop2^+^ clusters had significantly increased (Fig. 4B,C). At day 5, HE-T glands had recovered a normal architecture and contained a large number of Trop2^+^ clusters, the size of which had further increased in both corpus and antral glands (Fig. 4B-D). Consistent with an ongoing regeneration process, the number of proliferating cells per gland was higher in HE-T than in controls at all time points investigated, which is likely to reflect the effort to maintain a constant gland depth in damaged glands (Fig. 4E left, Fig. S4D). To test for potential involvement of Trop2^+^ cells in this process, Ki67/Trop2 co-staining was performed. At day 2, a high proliferation rate was observed in Trop2^+^ cells, and this increased further at later times (Fig. 4E right), indicating that the majority of Trop2^+^ cells are actively cycling.

We also explored whether expression of Trop2 may be induced in another type of injury, involving extended general tissue damage. For this purpose, adult mice were injected with indomethacin, an anti-inflammatory drug known to induce gastrointestinal ulcers in humans and in experimental animals (Anthony et al., 2000; Sun et al., 2015) (Fig. 4F). Histological examination of the stomach revealed the presence of mucosal/submucosal lesions in all treated mice, whereas no lesions were found in vehicle-treated mice (Fig. 4F). A significant induction of Trop2 expression was detected in indomethacin-treated stomach, in both antral and corpus glands, with the number of Trop2^+^ clusters appearing to be positively correlated with the extent of injury (Fig. 4F,G, Fig. S4E). As in the *Lgr5*-DTR model of injury, a high proportion (32%) of Trop2^+^ cells were in a proliferative state (Fig. 4H). These data suggest that re-expression of the Trop2 marker might be commonly associated with regeneration processes taking place in the stomach in response to epithelial injury.

### The origin of reactive adult Trop2^+^ cells

**Fig. 5.**
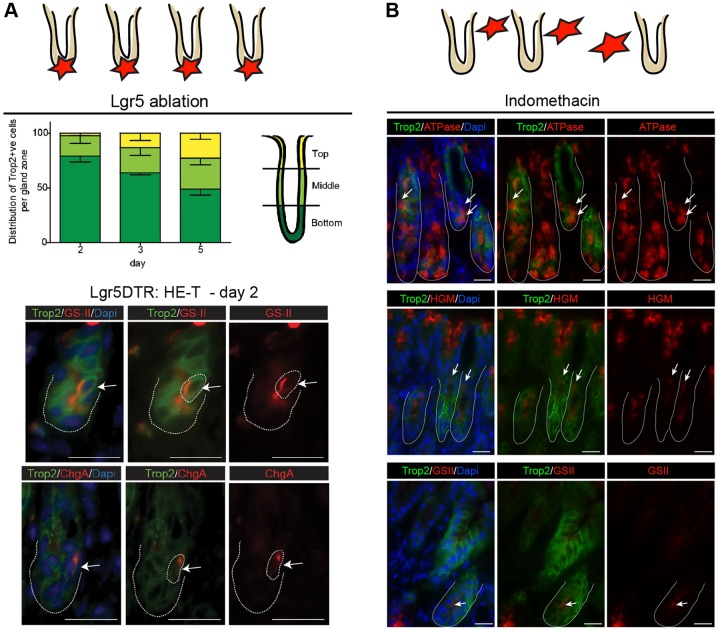
**Cells at the origin of reactive adult Trop2^+^ cells.** The two experimental models of injury are illustrated at the very top: the *Lgr5*-DTR model of localized specific ablation of the stem cell population (A) and the indomethacin-induced injury model that leads to extended damage throughout the epithelium (B). (A) (Top) Spatial distribution of Trop2^+^ cells after Lgr5^+^ cell ablation along antral glands (subdivided into top, middle and bottom zones, as depicted). Mice: *n*=3 at day 2 and 3, *n*=4 at day 5. (Bottom) Immunofluorescence showing co-expression of differentiation markers in Trop2^+^ cells of HE-T glands at day 2. Arrows indicate double-positive cells. Dotted lines outline gland limits and double-positive cells. (B) Immunofluorescence showing co-expression of differentiation markers in Trop2^+^ cells from indomethacin-treated mice. Arrows indicate double-positive cells. Scale bars: 20 µm.

In the absence of tools allowing direct tracing approaches, we attempted to identify the cells at the origin of Trop2^+^ cells in the two experimental models of injury. In the *Lgr5*-DTR model of localized specific ablation of the stem cell population, the distribution of reactive Trop2^+^ cells along the gland was first analyzed over time. Most cells were localized deep in the glands at day 2, whereas they were found throughout the glands at later stages, indicating that Trop2 is initially expressed by cells close to the stem cell zone (Fig. 5A). Just after damage induction (day 2), rare cells were identified in HE-T glands that co-expressed Trop2 and ChgA or the mucus neck-binding GS-II lectin (Fig. 5A), suggesting that Trop2 expression can initially occur in some differentiating or terminally differentiated cells belonging to the endocrine or mucous cell lineages. In the indomethacin-induced injury model, which leads to extended damage throughout the epithelium, Trop2 re-expression occurred in differentiated cells from the parietal and mucous cell lineages throughout the gland (Fig. 5B). Altogether, these observations suggest that proliferating Trop2^+^ cells might originate from differentiated epithelial cells located close to the damaged area. The possibility of an additional origin from another, as yet uncharacterized cell type cannot be excluded.

### Characterization of reactive adult Trop2^+^ cells upon Lgr5^+^ stem cell ablation

**Fig. 6.**
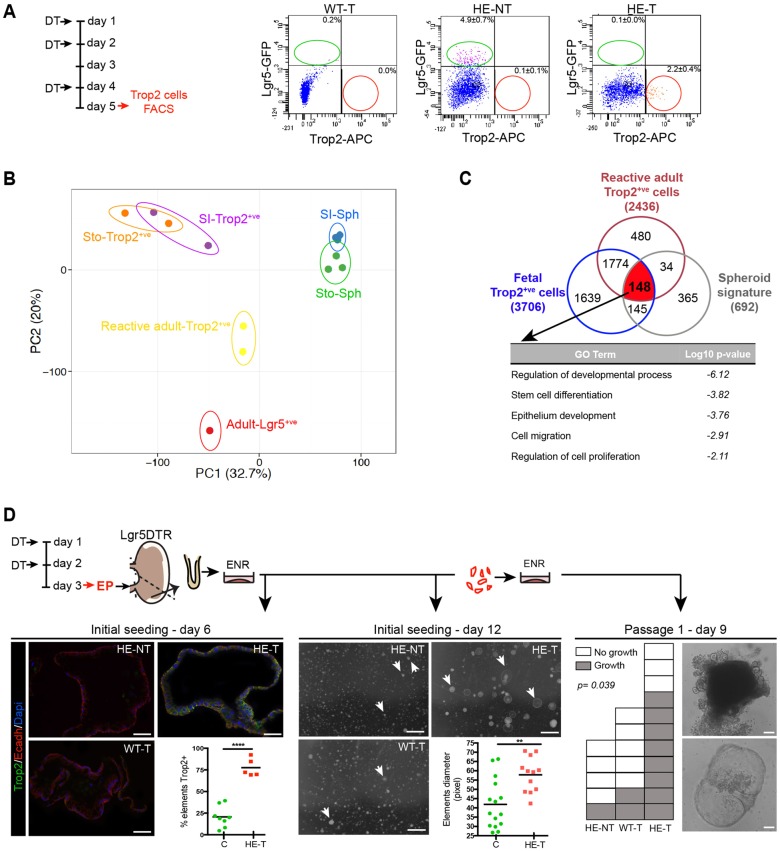
**Characterization of reactive adult Trop2^+^ cells following Lgr5^+^ stem cell ablation.** (A) Trop2^+^ cell isolation by FACS after DT treatment of *Lgr5*-DTR mice. (Left) Experimental scheme. (Right) Representative FACS plots. Percentages of Lgr5***^+^*** (green oval) or Trop2***^+^*** (red circle) cells per gate are shown as mean±s.e.m. for treated wild-type (WT-T; *n*=1), non-treated heterozygous (HE-NT; *n*=4) and treated heterozygous (HE-T; *n*=4) mice. (B) PCA plot of the whole transcriptome (19,468 genes) from sorted adult and fetal cells and fetal-derived spheroids. See legend to Fig. 2A. (C) Venn diagram showing overlap between ≥4-fold upregulated genes (number of genes in parentheses) in reactive adult and fetal Trop2^+^ cells versus Lgr5^+^ cells, with the fetal spheroid signature as defined in Fig. 2B. GO term analysis is shown for the genes common to the three lists. (D) *Ex vivo* culture of *Lgr5*-DTR antral glands after *in vivo* DT treatment. Control (C) mice are WT-T and HE-NT. Individual C and HE-T mice are represented by green circles and red squares, respectively. (Top) Experimental design. (Left) Representative images of immunofluorescence showing Trop2 expression at day 6 and quantification as a percentage of growing elements. Unpaired *t*-test, ****P=0.0001. (Center) Representative images of growing elements at day 12 after initial seeding of antral glands. Arrows point to surviving elements. Quantification of the mean diameter of elements/animal is shown. Unpaired *t*-test, **P=0.0017. (Right) Survival of replated elements at passage 1, day 9. Representative images are shown of elements grown from HE-T glands. Each square corresponds to an individual mouse; empty and filled squares represent no growth and growth, respectively. Fisher's exact test. Scale bars: 20 µm, left; 1 mm, center; 100 µm, right.

We used the *Lgr5*-DTR injury model to isolate antral reactive adult Trop2^+^ cells and to perform RNA-Seq analyses (Fig. 6A). PCA on the whole transcriptome indicated that emerging Trop2^+^ cells exhibit a unique pattern as compared with adult Lgr5^+^ stem and fetal Trop2^+^ cells (Fig. 6B). Nevertheless, using the transcriptome of adult Lgr5^+^ cells as a common reference, 79% (1922/2436) of the transcripts upregulated in reactive adult Trop2^+^ cells were commonly upregulated in fetal Trop2^+^ cells, indicating partial expression of overlapping genetic programs in regenerating adult and fetal-derived Trop2^+^ cells (Fig. 6C). Of relevance, 21.4% of the genes (148/692) constituting the fetal spheroid signature (defined in Fig. 2B and Table S1) were upregulated in both regenerating adult and fetal Trop2^+^ cells. GO term analysis of these common genes indicated high correlation with processes related to the regulation of development, stem cell differentiation, cell migration and proliferation (Fig. 6C). Of note, reactive adult as well as fetal Trop2^+^ cells did not show any evidence of transcript enrichment of adult stem cell/progenitor markers as compared with Lgr5^+^ cells, including the *Lgr5* gene itself (Fig. S5A, Table S2). Moreover, RNA-Seq analysis revealed differential expression of the Shh and Ihh ligands, as well as Ereg and Areg ligands, together with their cognate receptors, in adult and fetal Trop2^+^ cells as compared with Lgr5^+^ cells; this suggests the potential involvement of these signaling pathways in reactive Trop2^+^ cells (Table S2). Overall, transcriptome analysis indicated that adult Trop2^+^ cells involved in epithelial regeneration express genes that are part of a fetal developmental program.

Finally, the *ex vivo* growth properties of regenerating cells were studied by seeding antral glands from *Lgr5*-DTR HE-T or controls in Matrigel in ENR medium (Fig. 6D). At day 6, compared with controls, the majority (77.6±4.6%) of HE-T elements were composed of Trop2^+^ cells. At day 12, spheroid-like surviving elements were of a diameter that was statistically greater in HE-T than in control glands (Fig. 6D). Upon replating, HE-T samples also exhibited higher survival capacity than controls which, in agreement with Barker et al. (2010) and our own observations (Fig. 1D), show limited capacity to grow in ENR medium (Fig. 6D). However, after replating, not all adult-derived elements retained the spheroid-like shape, and some surviving elements evolved into organoid-like structures with protrusions (Fig. 6D). Despite maintaining Trop2 expression at levels similar to the fetal spheroids, adult-derived replated elements expressed pit and neck mucus differentiation markers (Fig. S5B). Together, these data indicated that, upon injury, emerging Trop2^+^ cells show a growth advantage compared with adult stem cells cultured under ENR conditions. Nonetheless, adult Trop2^+^ cells do not exhibit a stable spheroid-like phenotype like that of fetal cells, which is likely to reflect an incomplete fetal re-expression program.

## DISCUSSION

In the present study, we have shown that Trop2 marks fetal gastric epithelial cells and is re-expressed, together with other fetal markers, in cells contributing to regeneration of the glandular stomach following epithelial injury. We identified two kinds of Trop2^+^ cells in the fetal stomach generating two types of elements when grown *ex vivo* under the same culture conditions. Trop2^+^ cells isolated from the proximal stomach gave rise to circular multilayer organoids of squamous type, similar to those reported in human and mouse adult esophagus (Barbera et al., 2015; Jeong et al., 2015). By contrast, Trop2^+^ cells isolated from the pre-glandular epithelium generated hollow spheroids expressing low levels of gastric markers despite definite commitment to a gastric fate, as shown when cultured *ex vivo*. Whereas Trop2 expression persisted in the postnatal and adult periods in the squamous portion of the mouse stomach, Trop2^+^ cells were only transiently present in the fetal presumptive glandular stomach. Progressive loss of Trop2 coincided with the onset of gland formation and concomitant cell lineage differentiation, as well as with loss of spheroid formation capacity *ex vivo*. A similar observation has been reported for the related Trop2^+^ intestinal progenitors isolated from E14.5-16.5 fetus (Mustata et al., 2013). This supports the notion that the poorly differentiated spheroids grown from fetal stomach and intestine represent ‘frozen’ states of embryonic cells displaying commitment to their respective fate.

Interestingly, despite the expected distortion of the transcriptome in Trop2^+^ cells induced by *ex vivo* culture, fetal Trop2^+^ cells and cultured Trop2^+^ spheroids share expression of embryonic markers and similarly express lower levels of Lgr5 than adult glandular stem cells, which fits with the low Wnt activity reportedly associated with gastric development *in vivo* (Sherwood et al., 2011). In this regard, our immunofluorescence and lineage-tracing experiments suggest that the glandular epithelium of the stomach might be generated in two waves. The first, which depends on early Cnx43^+^ embryonic epithelial cells, generates mainly the fetal pre-glandular epithelium, but also contributes to a proportion of the postnatal epithelium. A second wave, relying on later, highly proliferative Cnx43^−^ progenitors, generates the bulk of the postnatal epithelium, leading to the progressive dilution of the Cnx43-traced clones. Future studies will be needed to determine the precise nature of these later progenitors, with Sox2^+^ and Lgr5^+^ cells among the potential candidates (Arnold et al., 2011; Barker et al., 2010).

Recently, it has been reported that mature adult glandular stomach can be obtained from human iPSCs and mouse ESCs in a process of specification and maturation that takes no less than 34 days (McCracken et al., 2014; Noguchi et al., 2015). The present study shows the possibility of converting undifferentiated spheroids obtained from E14.5 fetal stomach into organoids containing the differentiated cell types of the adult glands. The convenience of the isolation and maintenance procedures employed to culture Trop2^+^ fetal cells presents an advantage in this alternative *ex vivo* system, which may be exploited to further dissect the mechanisms involved in the primary to secondary transition stages that occur in the glandular stomach.

In adults, the regeneration capacity of a tissue has been correlated with the presence of undifferentiated stem cells or with the plasticity of differentiated cells. In lower vertebrates, differentiated cells can revert to a fetal-like stage with high proliferative capacity to repair damaged tissue. Thereafter, redifferentiation into the adult cell types allows restoration of full tissue functionality (Singh et al., 2015). Lineage-tracing approaches in the mouse have revealed that a similar process of de-differentiation can occur during epithelium repair in mammals. Committed secretory cells can acquire a stem-like state upon airway stem cell ablation in lungs or sublethal irradiation in the intestine (Tata et al., 2013; van Es et al., 2012). However, the transcriptome of the de-differentiated cells was not investigated. In the present study, we provide evidence that a similar process takes place in the stomach following Lgr5^+^ stem cell ablation or an acute injury secondary to indomethacin administration. Within 24 h after initiation of the damage, some differentiated cells started to express Trop2, and the majority of reactive Trop2^+^ cells entered the cell cycle. A contribution of other cell types to the origin of Trop2^+^ cells is presently not formally excluded. Among potential candidates are Tnfrsf19^+^ cells, previously described as a corpus reserve stem cell pool in proliferation-depleted glands (Stange et al., 2013). Their involvement seems, however, unlikely since the kinetics of Tnfrsf19^+^ cell-dependent regeneration was reportedly much slower, only taking place after 1 week. In addition, unlike Tnfrsf19^+^ cells, reactive Trop2^+^ cells are prevalent in the antrum and show lower levels of Tnfrsf19 expression than Lgr5^+^ stem cells.

Our RNA-Seq data demonstrated that re-expression of the Trop2 marker in adults is the hallmark for a global change in the expression profile, shifting towards a fetal-like expression pattern, similar to that of fetal Trop2^+^ or spheroid-generating cells. This is characterized by a robust induction of the whole proliferation machinery, coherent with a high proportion of Trop2^+^ cells entering the cell cycle and the expression of genes associated with tissue morphogenesis and organ development. Comparison of the signaling pathways between reactive adult and fetal Trop2^+^ cells and resident Lgr5^+^ stem cells revealed active involvement of the Hedgehog pathway, which is well known in lower vertebrates to contribute to tissue regeneration (Singh et al., 2015). Moreover, the Areg/Ereg cascades, which are reported to participate in epithelial regeneration, might also take part in cell signaling in adult Trop2^+^ cells, as opposed to Lgr5^+^ stem cells (Liu et al., 2012; Nagai et al., 2014).

Trop2 re-expression has also been reported in other regeneration processes in adult mice: in the liver, upon diethoxycarbonyl dihydrocollidine (DDC) diet injury, and in the prostate, where Trop2^+^ cells can regenerate prostatic tubules *in vivo* (Goldstein et al., 2008; Okabe et al., 2009). Regarding the fate of the activated Trop2^+^ cells, it has been shown in the DDC model that the clonally expanded regenerating cells have the capacity to differentiate into hepatocytes and cholangiocytes (Okabe et al., 2009). In the present study, the toxicity observed in DT-treated *Lgr5*-DTR heterozygous mice and the lack of tools allowing long-term tracing of Trop2^+^ cells did not permit us to fully explore their differentiating capacity. However, the observation that *ex vivo* cultured adult Lgr5-depleted glands (i.e. those depleted of Lgr5^+^ stem cells) exhibited signs of differentiation into adult epithelial lineages suggests that, *in vivo*, regenerating Trop2^+^ cells have the capacity to redifferentiate into the adult cell lineages after tissue repair.

Although the function of Trop2 is still unclear, it is overexpressed in many cancers, where it plays a role in regulating cell growth and migration (Fornaro et al., 1995; McDougall et al., 2015; Shvartsur and Bonavida, 2015). In the present study, the Trop2^+^ cells emerging along the gastric glands after stem cell ablation are highly proliferative and mainly undifferentiated, showing re-acquisition of fetal characteristics. Future studies are needed to determine whether Trop2 must be considered as a simple marker of embryonic and regenerating cells or plays a functional role in gastric epithelial morphogenesis during development and in adults during regeneration.

## MATERIALS AND METHODS

### Mice

Animal procedures complied with the guidelines of the European Union and protocols were approved by the local ethics committee of the Medicine Faculty, Université Libre de Bruxelles (CEBEA). Mouse strains were CD1 (Charles River), Cnx43-KI-CreER(T) (European Mouse Mutant Archive), Rosa26R-YFP and Rosa26R-Tomato (both from The Jackson Laboratory). *Lgr5*-DTR-EGFP knock-in mice were kindly provided by Genentech (Tian et al., 2011). The day the vaginal plug was observed was considered as E0.5.

For lineage-tracing experiments, tamoxifen (Sigma-Aldrich) was dissolved in sunflower oil (Sigma-Aldrich)/ethanol mixture (9:1) at 10 mg/ml. Pregnant females were injected intraperitoneally at 0.1 mg/g body weight. For specific cell ablation in *Lgr5*-DTR mice, diphtheria toxin (Sigma-Aldrich) was injected intraperitoneally (50 µg/kg) into 8-week-old mice. Control mice were injected with sterile PBS solution. Eight-week-old CD1 mice were injected subcutaneously with 300 µg/kg indomethacin (dissolved in 0.6 M NaHCO_3_ buffer pH 8.5 and 5% DMSO) or with the buffer as control.

### Histology and immunostaining

Dissected stomachs or *ex vivo* cultured spheroids and organoids were fixed with neutral-buffered 10% formalin solution (Sigma-Aldrich) and sedimented through 30% sucrose solution before OCT (Tissue-Tek) embedding. Histological protocols and immunofluorescence/immunohistochemistry experiments were carried out as previously described (Garcia et al., 2009). Primary antibodies are detailed in the supplementary Materials and Methods. The Alexa Fluor 647-conjugated GS-II lectin was obtained from Molecular Probes (L-32451) and the *in situ* cell death TUNEL detection kit from Roche. Samples were visualized using a Zeiss Axioplan 2 or Zeiss Observer Z1 microscope. Quantifications are detailed in the supplementary Materials and Methods.

### Flow cytometric analysis and cell sorting (FACS)

Embryonic stomach samples or adult *Lgr5*-DTR antral glands, isolated as previously described (Barker et al., 2010), were dissociated with the StemPro Accutase cell dissociation reagent (Thermo Electron) and passed through a 40-μm nylon cell strainer (Greiner).

Fluorochrome-conjugated antibodies or relevant isotype controls were used for staining in PBS containing 2% BSA and 2 mM EDTA for 45 min on ice and sorted using a FACSAria I (BD Biosciences). For spheroid formation efficiency measurements, sorted cells were cultured *ex vivo* under ENR conditions. Quantification methods are detailed in supplementary Materials and Methods. For RNA-Seq analysis, *Lgr5*-DTR-EGFP^+^ cells were directly sorted using the FITC channel. Sorted cells were directly collected for RNA-Seq analysis over Qiazol lysis reagent (Qiagen). For adult Trop2^+^ and Lgr5-GFP^+^ sorting, cells were pooled (two pools of two HE-T samples, one pool of four HE-NT samples) to extract RNA from a total of 4000-8000 cells. For fetal Trop2^+^ cells, a mean of 30,000 cells were sorted in each independent experiment.

### *Ex vivo* culture

Embryo stomach and small intestine were dissociated as reported (Mustata et al., 2013) and cultured according to the protocol reported (Sato et al., 2009). Specifically, the culture medium used for growing gastric spheroids (ENR) comprised a basal medium [Advanced DMEM/F12 supplemented with 2 mM L-glutamine, N2 and B27 without vitamin A (Invitrogen), gentamycin, penicillin-streptomycin cocktail, 10 mM HEPES, 1 mM N-acetyl cysteine] supplemented with growth factors at a final concentration of 50 ng/ml EGF and 100 ng/ml noggin (both from Peprotech) and 100 ng/ml CHO-derived R-spondin 1 (R&D Systems). Culture medium was changed every other day, and after 5-6 days in culture the spheroids were harvested, mechanically dissociated and replated in fresh Matrigel (BD). The production of fetal gastric spheroids, as well as repeated replatings, were performed in six independent culture experiments, starting from either individual embryos or pools of embryos.

Adult antral glands from *Lgr5*-DTR mice were isolated and cultured as reported (Barker et al., 2010). Culture medium used for adult-derived gastric organoid growth (ENRFGW) comprised basal medium (see above) supplemented with growth factors at a final concentration of 50 ng/ml EGF, 100 ng/ml noggin, 200 ng/ml CHO-derived R-spondin 1, 100 ng/ml Fgf10 and 100 ng/ml Wnt3a (both R&D Systems), 10 nM gastrin (Sigma-Aldrich). Media were supplemented with 10 µM Y-27632 (Sigma-Aldrich) in all initial seeding and replating experiments. Images were acquired with a Moticam Pro camera connected to a Motic AE31 microscope or with a Leica DFC 420C camera using Leica Application Suite V3.8 software. Further details of *ex vivo* culture are provided in the supplementary Materials and Methods.

### Gene expression analysis

qRT-PCR was performed on total RNA as reported (Garcia et al., 2009). Expression levels were normalized to that of the reference genes *Rpl13* and *Gapdh*. Gastric and intestinal spheroid samples were obtained from independent pools of E15-16 embryos, and organoid samples were obtained from E16 embryos and adult mice. Each sample was run in duplicate. Primer sequences are provided in the supplementary Materials and Methods.

### RNA-seq and transcriptome analysis

RNA from spheroids/organoids and from sorted cells was extracted using the miRNA isolation kit (Ambion, Life Technologies, AM1560) and with the miRNeasy Micro Kit (Qiagen), respectively. RNA quality was checked by Bioanalyzer (Agilent). Indexed cDNA libraries were prepared using the TruSeq Stranded mRNA sample preparation kit (Illumina) and the Ovation single-cell RNA-Seq system (NuGEN) for RNA extracted from spheroids/organoids and sorted cells, respectively. RNA-Seq and transcriptome analysis methods are detailed in the supplementary Materials and Methods. Transcript profiling data are available at GEO under accession number GSE65395.

### Statistical analysis

Statistical analyses were performed with Graph Pad Prism 6. All experimental data are expressed as mean±s.e.m. The tests used to determine statistical significance of differences between groups are indicated in the figure legends.
